# Identification of Key Residues Essential for the Activation of Plant Immunity by Subtilisin From *Bacillus velezensis* LJ02

**DOI:** 10.3389/fmicb.2022.869596

**Published:** 2022-08-15

**Authors:** Jianan Hu, Ruokui Chang, Yujin Yuan, Zhuoran Li, Yuanhong Wang

**Affiliations:** ^1^College of Horticulture and Landscape Architecture, Tianjin Agricultural University, Tianjin, China; ^2^College of Engineering and Technology, Tianjin Agricultural University, Tianjin, China

**Keywords:** *Bacillus velezensis* LJ02, subtilisin point mutant, systemic acquired resistance, PAMP-triggered immunity, *Botrytis cinerea*

## Abstract

Subtilisin, a serine protease, can trigger defense responses in a wide variety of plants, both locally and systemically, to protect against pathogens. However, key residues of subtilisin to improve resistance to plant diseases remain unknown. In this study, *Nicotiana benthamiana* (*N. benthamiana*) leaves expressing subtilisin from *Bacillus velezensis* LJ02 were shown to improve protection against *Botrytis cinerea* (*B. cinerea*). Furthermore, the underlying mechanism that LJ02 subtilisin improved the protective effect was explored, and the direct inhibitory effect of subtilisin on *B. cinerea* was excluded *in vitro*. Subsequently, reactive oxygen species (ROS) burst and upregulation of resistance-related genes in systemic leaves of *N. benthamiana* further verified that subtilisin could induce systemic protection against *B. cinerea*. G307A/T308A and S213A/L214A/G215A subtilisin significantly reduced the ability to resist *B. cinerea* infection in *N. benthamiana*. Furthermore, the ROS content and expression levels of resistance-related genes of both mutants were significantly decreased compared with that of wild-type subtilisin. This work identified key residues essential for the activation function of subtilisin plant immunity and was crucial in inducing plant defense responses against *B. cinerea*.

## Introduction

*Botrytis cinerea* (*B. cinerea*), a necrotrophic fungal pathogen, is the causal agent of blight, rot and gray mold in more than 1000 plant species (Fillinger and Elad, 2016), including almost all economically important vegetables, fruits and crops, and annually causes huge economic losses worldwide (Weiberg et al., 2013). Gray mold is prone to occur prior to harvest, or even at the seedling stage in some plants. In severe cases, the incidence of gray mold in plants can reach 100% (Wang et al., 2019), resulting in massive losses of plants seedlings. *Bacillus velezensis* (*B.velezensis*) such as CE100 (Choi et al., 2020), XT1 (Torres et al., 2020), and LJ02 (Li et al., 2015), as potential and efficient agents demonstrated robust biocontrol activity against *B. cinerea*. *B. velezensis* can encode immune proteins that can be recognized like the perception of a pathogen by plant to induce disease resistance, which has attracted wide attention, and thus has the advantage of triggering sophisticated and effective defense responses. It was found that flagellin from *B. velezensis* LJ02 can stimulate defense responses and increase resistance to *Nicotiana tabacum var*. *Xanthi* to Tobacco mosaic virus (TMV) (Wei et al., 2021). Further studies showed that PeBA1 of *B. amyloliquefaciens* NC6 induced resistance in *Nicotiana tabacum* against TMV (Wang et al., 2016). However, there are limited reports that immune proteins secreted by *B. velezensis* can enhance plants disease resistance to *B. cinerea*.

Plant recognition of pathogen-associated molecular pattern (PAMP) triggers a defense response known as PAMP-triggered immunity (PTI), which activates a cascade of signaling events that culminate in immune responses, including ion fluxes, activation of mitogen-activated protein kinases (MAPK), and the production of reactive oxygen species (ROS; Vatsa et al., 2011; Ngou et al., 2021). Bacterial PAMPs not only play an important role in basal resistance to pathogens but also contribute to the induction of systemic-acquired resistance (SAR; Mishina and Zeier, 2007). SAR, which often develops in uninfected areas of plants, is a systemic protection against other infections that gradually spread throughout the plant, and usually develops a long-lasting improved resistance to further attacks by pathogens (Gao et al., 2015). Recognition of PAMP recognition initiates the MAPK signaling cascade, one of the earliest signaling events consisting of the first step in PTI (Nie et al., 2017), and activates WRKY transcription factors (e.g., WRKY7/WRKY8), resulting in the upregulation expression of resistance-related genes (Zhong et al., 2018). PTI is accompanied by a set of induced responses that usually repel pathogen attacks, such as activation of PTI marker genes *ACRE31* (Ma et al., 2021) and *FRK* (Wen et al., 2021). The ROS burst is believed to act downstream of PAMP/pathogen-responsive MAPKs (Yoshioka et al., 2003). Pti1 serine-threonine kinase acts early in PTI by inducing ROS production in response to perception of PAMP (Schwizer et al., 2017). ROS contributes to the restriction of further infection by pathogens, direct attack on pathogens, and triggering SAR (Alvarez et al., 1998; Torres et al., 2006). The rapidly generated *Avr9/cf-9* genes (Acre) are candidates for components of signaling pathways involved in the activation of later defense responses in PTI. *ACRE 31*, a putative calcium-binding protein, can be rapidly induced by the elicitor and involved in the plant defense signaling cascade (Durrant et al., 2000).

Endogenous plant proteases are essential in many aspects of plant immunity (Rawlings et al., 2014), among which serine proteases are the most abundant in plants (Clemente et al., 2019). Subtilisins of the S8 family are one of the main serine proteases and have shown multiple roles in defence responses, ranging from immune priming to the activation of resistance-related genes, the generation of antimicrobial peptides, and the recognition or processing of pathogen effectors (Balakireva and Zamyatnin, 2018). Due to the large number of duplications, losses, and functional diversifications in the evolution of subtilisin superfamily, there are large differences in subtilisin phylogeny (Li et al., 2017). Subtilisin has a certain degree of conservation across different classes of microbes to general microbial fitness (Caro et al., 2020), while conserved functional residues are critically important for protein function. For example, specific residues of AtZAR1, a canonical CC-type NLR protein from *Arabidopsis*, are required for its immune function against *Pseudomonas syringae* pv. *tomato* DC3000 (Baudin et al., 2017). In search of whether the proteolytic activity of AsES, a subtilisin from *Acremonium strictum*, is required to trigger the defence response, its mutants at the active site (S226A) have confirmed that AsES induced the plant defense, and that enzymatic and eliciting activities were not associated (Caro et al., 2020). However, key residues of subtilisin triggering defence responses to pathogens remain largely unknown.

In this study, we focused on subtilisin secreted by *Bacillus velezensis* LJ02. Previous studies have shown that subtilisin can induce SAR and confer resistance against *Botrytis cinerea* in plants (Hael-Conrad et al., 2018). However, key residues of subtilisin essential for activation of plant defense responses were not yet identified. In this study, we verified the protective effect of subtilisin from LJ02 against *B. cinerea* by transient expression in *Nicotiana benthamiana*. ROS burst and upregulated expression of resistance-related genes showed that subtilisin can induce plant defense responses. Multiple alignment analysis of subtilisin revealed the conserved amino acids of subtilisin in different strains. We performed comparative analysis and mutation of conserved residues and expressed subtilisin and its mutants in *N. benthamiana*. Moreover, we measured ROS accumulation and resistance-related gene expression levels of systemic leaves to identify residues with key roles in subtilisin-activated plant defense responses.

## Materials and Methods

### Pathogen and Plant Culture

*Nicotiana benthamiana* was grown in an INE800 Memmert incubator at a temperature of 25°C in a 16 h light/8 h dark cycle for 4 weeks after sowing. *B. velezensis* LJ02 and *B. cinerea* were provided and preserved by the Plant Immunity and Biological Control Laboratory, Tianjin Agricultural College.

### Subtilisin Protein Prokaryotic Expression and Purification

Genomic DNA from *B. velezensis* LJ02 was isolated using the Bacterial Genomic DNA Extraction kit (Solarbio). The subtilisin coding sequence (Supplementary Data 1) was cloned using genomic DNA from *B. velezensis* LJ02. Both amplified subtilisin fragments and prokaryotic expression vector pET-28a were ligated by Rapid DNA Ligation kit (Roche). Constructed pET-28a-subtilisin plasmid were transformed into the *Escherichia coli* BL21(DE3). The competent cells with OD_600_ of 0.6 were induced with isopropyl β-D-1-thiogalactopyranoside (IPTG) at 16°C for 14 h. Cells were harvested by centrifugation for 30 min at 4°C. Supernatants were removed and analyzed by 12% sodium dodecyl sulfate-polyacrylamide gel electrophoresis (SDS-PAGE). Cells were suspended in 10 ml of lysis buffer (50 mM Tris-HCl (pH 8.0), 300 mM NaCl, and 20% glycerol (w/v) prior to disruption at 4°C by ultrasonication (3 min × 1 min). Cell debris was pelleted by centrifugation for 1 h at 4°C. Supernatants were applied onto Ni Sepharose 6 Fast Flow (Cytiva) for purification. Recombinant subtilisin proteins were analyzed in 12% SDS-PAGE stained with Coomassie Brilliant Blue (Solarbio).

### Western Blot

Proteins were extracted from plants as described by Han et al. (2022). Western blots were performed by SDS-PAGE with the Mini-PROTEA^§^ Tetra System (BIO-RAD). Proteins were transferred to the polyvinylidene fluoride (PVDF) membrane with the TransBlot turboTM system (Bio-rad), and the membrane was blocked for 2 h with 1 × Tris-buffered saline (TBS) containing skim milk (0.5%) followed by 2 h incubation with primary antibody. After washing with TBS, the membrane was incubated with the secondary antibody for 1 h, followed by wash with TBS. The photographic developer was sprayed (A solution + B solution mix at 1:1) onto the membrane. The primary antibody was Anti-DYKDDDDK Monoclonal Antibody (TRANS), Actin mouse anti-antibody (Bioss), and the secondary antibody was Goat Anti-Mouse IgG, HRP (TRANS). The blot was recorded using Tanon 5200 Multi.

### Subtilisin Protease Assay

As described previously by Kobayashi et al. (1995) and Jaouadi et al. (2012), subtilisin protease activity was measured with N-succinyl-Ala-Ala-Pro-Phe-*p*-nitroanilide (Suc-AAPF-*p*NA; 2 mg/ml in 50% dimethyl formamide) as substrates. One hundred microliters of enzyme prepared in 50 mM Tris–HCl buffer (pH 8.0), containing 5 mM CaCl_2_ at a concentration of 8.25 mg/ml, were added to 20 ml of substrate. After incubation at 37°C for 2 h, the amount of *p*-nitroaniline released was measured by monitoring the absorbance at 405 nm. A sample blank without substrate was routinely included. One unit (U) of enzymatic activity was defined as the amount of enzyme that liberated 1 μmol of *p*-nitroaniline per minute under the conditions of the assay.

The activity of the subtilisin protease was measured at pH values ranging from 3 to 11 using Nsuc-AAPF-*p*NA as the substrate. The following 50 mM buffers containing 5 mM CaCl_2_ were used: (pH 3.0–5.0), Tris–HCl (pH 6.0–8.0), and carbonate (pH 9.0–11.0). The effect of incubation temperature (25, 30, 35, 40, and 45°C) and storage time (0, 1, 2, 3, and 4 days) on the degradation of Nsuc-AAPF-*p*NA by the subtilisin protease was evaluated.

### Effects of Subtilisin on *Botrytis cinerea*

All plates and materials were sterilized in an autoclave oven before experiments, and experimental operations were conducted in a sterile bench. The oxford cup method was conducted according to previously described method (Li et al., 2013). Antifungal activity was evaluated by the diameter of transparent circles around oxford cups.

*Botrytis cinerea* was induced to sporulate on potato dextrose agar (PDA) medium. Spores were scraped from the agar plates and then made into a spore suspension (30–100 spores per field of view under low power). Spores Germination Assays were performed according to the method of Maribel (Plascencia-Jatomea et al., 2003). The number of spore germination was checked under the microscope (the length of the germ tube was longer than the short radius of the spore was regarded as germination) after 12 h. The inhibition rate of spore germination was calculated according to the method described previously (Zhao et al., 2014).

Subtilisin was infiltrated into the lower leaves of *N. benthamiana* by needleless syringe. Upper systemic leaves of *N. benthamiana* were collected and inoculated with mycelial discs of *B. cinerea* at 3 days after infiltrated (DAI; Wang et al., 2016). The inoculated leaves are stored in a humid chamber. The lesions’ diameter was measured using the criss-cross method (Ji et al., 2014) and then photographed.

### Sequence Alignment, Classification, and Phylogenetic Tree Analysis

Totally, 20 top organisms of subtilisin amino acid sequences by taxon were downloaded from NCBI (Supplementary Data 2) and analyzed using DNAMAN for multiple alignments to identify and present conserved residues. The evolutionary history was inferred using the UPGMA method (Sneath and Sokal, 1973). The optimal tree is shown. The evolutionary distances were computed using the Poisson correction method and are in the units of the number of amino acid substitutions per site. Poorly aligned positions and divergent regions of MSA were eliminated using Gblocks Version 0.91b with a less stringent setting to make it more suitable for phylogenetic analysis. Then, a neighbor-joining (NJ) tree was constructed using MEGA 11 with 1,000 of bootstrap replications, pairwise deletion, and Poisson model.

### Transient Expression of Subtilisin and Its Mutants in *Nicotiana benthamiana*

The subtilisin gene was amplified and homologously recombined with the pK7WG2D (a transient expression vector, expressed by 35S promoter) by using the ClonExpressII One Step Cloning Kit (Vazyme Biotech). The point mutation was introduced into the subtilisin DNA fragments by fusion PCR method with primers bearing desired point mutation. In total, 13 coding sequences of subtilisin mutants were amplified and cloned into vector pK7WG2D. The primer sequences and vectors used for the plasmid constructs are shown in Supplementary Table 4. The constructed vectors were transformed into *Agrobacterium* GV3101. *Agrobacterium*-mediated transient expression was performed as described (Li Z. et al., 2019). The upper systemic leaves of *N. benthamiana* were collected and inoculated with *B. cinerea* mycelial discs at 3 DAI as previously described. The inoculated leaves were stored in a humid chamber. The lesions’ diameter were measured using the criss-cross-method (Ji et al., 2014) at 3 days after inoculation with *B. cinerea* and then photographed.

### ROS Measurement

The generation of ROS during subtilisin infiltration was determined using a commercial plant ROS enzyme-linked immnunosorbent assay (ELISA) kit (Chundubio). Three or four upper leaves without infiltration of *N. benthamiana* were harvested at various times after infiltration with subtilisin, 0.1 g was fully ground in liquid nitrogen, and phosphate-buffered saline (pH 7.4) was added. The plant tissue was homogenized with a low-temperature homogenizer, centrifuged twice for about 20 min, and then the precipitate was discarded for use in ELISA. The standard curve was used to determine the amount in each unknown sample. OD_450_ values were measured with an ELISA reader (Promega). Meanwhile, ROS generation was detected using 3,3′-diaminobenzidine (DAB) solution (Solarbio) as described previously (Yang et al., 2018). Leaves were then decolorized in boiling ethanol (90%) for 30 min and were photographed by camera (Zhao et al., 2018).

### RNA Extraction and Quantitative RT-PCR

*WRKY7, WRKY8, ACRE31, Pti1, CYP71D20*, and *FRK* primers in *N. benthamiana* genome database^1^ were designed using the SnapGene software. *Actin* (Tao et al., 2011) was used as the internal reference gene (Supplementary Table 5). The extraction of total RNA from *N. benthamiana* leave samples was carried out according to the instructions of TIANGEN RNAsimple Total RNA Extraction Kit. Using total RNA as a template, Takara Prime Script TM RT regent Kit with gDNA Eraser (Perfect Real-Time) was used for reverse transcription. The primers and reaction system were added according to the instructions (Takara). The 2^–ΔΔCt^ calculation method was used for analysis (Livak and Schmittgen, 2001).

## Results

### Subtilisin Improves the Protective Effect of *Nicotiana benthamiana* to *Botrytis cinerea*

**FIGURE 1:**
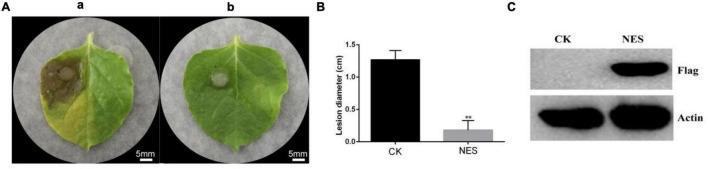
Subtilisin transient expression improves the control effect in *N. benthamiana* against *B. cinerea*. **(A)** Representative phenotypes of the disease caused by *B. cinerea* in *N. benthamiana* leaves expressing pK7WG2D (a) and pK7WG2D-subtilisin (b) at 3 DAI. Photographs were taken at 3 days after inoculation with *B. cinerea.* Experiments were carried out with five leaves per treatment. **(B)** Lesion diameter of disease caused by *B. cinerea* in *N. benthamiana* leaves expressing pK7WG2D (CK) and pK7WG2D subtilisin (NES). Data presented in **(B)** are the means ± SD of lesion diameter of five leaves. The statistical analyses were performed using the Student’s *t*-test; and asterisks indicate significant differences between pK7WG2D and pK7WG2D-subtilisin treatment (** *p* < 0.01). **(C)** Protein accumulation detected by western blot using anti-flag and anti-Actin antibody in *N. benthamiana* leaves expressing pK7WG2D (CK) and pK7WG2D subtilisin (NES).

The systemic leaves of *N. benthamiana* expressing pK7WG2D (CK) and *N. benthamiana* expressing pK7WG2D-subtilisin (NES) were inoculated with *B. cinerea*. A severe symptom was observed on the systemic leaves of CK compared with NES at 3 days after inoculation (Figure 1A), and the lesion diameter on the systemic leaves of NES was significantly smaller than that of CK (Figure 1B). Western blot analysis of NES leaves showed that the subtilisin protein was stably accumulated at 3 DAI (Figure 1C). In addition, the lesion diameter of the systemic leaves in *N. benthamiana* infiltrated with purified subtilisin protein was significantly smaller than that in *N. benthamiana* infiltrated with buffer, similar to the transient expression results (Supplementary Figure 1).

### Subtilisin Has No Direct Inhibitory Effect on *Botrytis cinerea*

**FIGURE 2:**
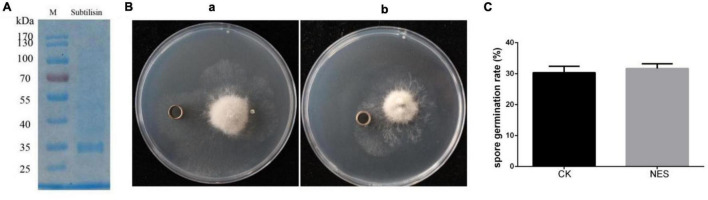
Antifungal effect of subtilisin *in vitro*. **(A)** sodium dodecyl sulfate-polyacrylamide gel electrophoresis (SDS-PAGE) image of purified subtilisin protein, Molecular mass markers (M) indicated on the left from top to bottom is 170, 130, 100, 70, 55, 40, 35, and 25 kDa. **(B)** Antifungal experiment of buffer (a) and purified subtilisin (b) *in vitro.* After *B. cinerea* was cultured on the potato dextrose agar (PDA) medium for 3 days, 100 μl of 100 μg⋅ml^–1^ subtilisin protein was added to the Oxford cup and placed in a 28°C incubator for 3 days, and then, the diameter of transparent circles around oxford cups was observed. **(C)** The effect of subtilisin on the *B. cinerea* germination. Data presented in **(C)** are the means ± SD from three independent experiments. The statistical analyses were performed using the Student’s *t*-test, and asterisks indicate significant differences.

Subtilisin has been shown to provide protection in *N. benthamiana* against *B. cinerea*. To further determine whether subtilisin itself has a direct antifungal effect against *B. cinerea*, subtilisin protein activity was measured, the purified subtilisin protein was used for bacteriostatic experiments (Figure 2A), and the experimental group with protein buffer was used as CK. The activity of subtilisin protein was relatively stable in PDA medium when incubated at 28°C for 3 days (Supplementary Figure 2), and there is no inhibition zone around the oxford cup with purified subtilisin (Figure 2B), indicating that subtilisin had no clear inhibitory effect on hyphal growth *in vitro*. Furthermore, the germination rate of spores inoculated with purified subtilisin did not show significant differences compared with that of CK (Figure 2C), suggesting that subtilisin does not have an effect on the germination of spores. Together, the hypothesis that subtilisin directly inhibits the growth of *B. cinerea* was ruled out.

### Subtilisin Stimulates the Immune Resistance of *Nicotiana benthamiana*

**FIGURE 3:**
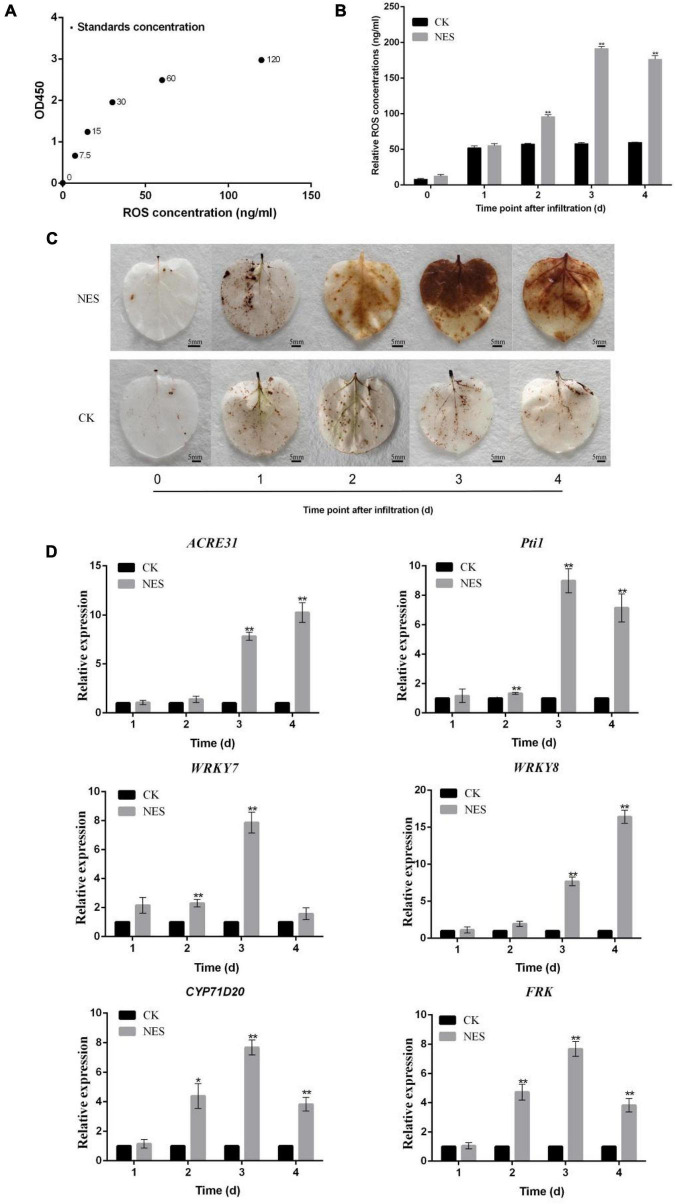
Changes in reactive oxygen species (ROS) and gene expression in response to subtilisin. **(A)** Standard curve for the determination of ROS concentration. The standard curve was generated by plotting the average OD_450_ of the six plant ROS standard concentrations (0, 7.5, 15, 30, 60, and 120 ng/ml) provided in the kit on the vertical axis (X) vs. the corresponding concentration on the horizontal axis (Y). **(B)** ROS concentration in *N. benthamiana* leaves expressing pK7WG2D (CK) and pK7WG2D-subtilisin (NES) measured by enzyme-linked immunosorbent assay (ELISA) at 1–4 DAI. **(C)** Accumulation of ROS was analyzed in *N. benthamiana* leaves expressing pK7WG2D (CK) and pK7WG2D-subtilisin (NES) at 1–4 DAI. ROS was visualized by 3,3′ diaminobenzidine (DAB) staining methods. Brownish deposits were indicative of ROS. **(D)** Expression analysis of resistance-related genes *WRKY7/8, ACRE31, Pti1, CYP71D20*, and *FRK* in *N. benthamiana* expressing pK7WG2D (CK) and pK7WG2D-subtilisin (NES) at 1–4 DAI. The samples were normalized against *Actin* and expression levels are represented as fold changes relative to the control. Data presented in **(D)** are the means ± SD of three independent experiments. The statistical analyses were performed using the Student’s *t*-test, and asterisks indicate significant differences (*p < 0.05; **p < 0.01).

Since subtilisin cannot inhibit the growth of hyphals and germination of *B. cinerea*, it implied that subtilisin may act by an effect on plant defense responses. To further verify this hypothesis, we characterized the defense responses induced by subtilisin in *N. benthamiana.* The standard curve was generated by plotting the average OD450 of ROS standard concentrations (Figure 3A) and the ROS accumulation and concentration in *N. benthamiana* expressing PK7WG2D (CK) and pK7WG2D-subtilisin (NES) at various times were analyzed. Compared with CK, ROS concentration (Figure 3B) and brown DAB-stained precipitates (Figure 3C) in NES were increased at 2–4 DAI, demonstrating that subtilisin infiltration of *N. benthamiana* can cause ROS accumulation, a typical phenotype of plant defense response.

Changes in expression pattern of resistance-related genes are important plant responses to PAMPs and pathogen (Meng and Zhang, 2013). Therefore, the expression levels of resistance-related genes *WRKY7, WRKY8, ACRE31, Pti1, CYP71D20*, and *FRK* were systematically investigated in CK and NES at 1–4 DAI. Compared with CK, the expression levels of *WRKY7* of NES all showed a significant increase at 2 DAI and 3 DAI. *ACRE31* and *WRKY8* expression levels of NES all showed significantly increased at 3 DAI and 4 DAI. *Pti1*, *CYP71D20*, and *FRK* expression levels of NES were significantly higher than those of CK at 2–4 DAI (Figure 3D). The expression of the resistance-related gene indicated that subtilisin is involved in the activation of plant defense responses in *N. benthamiana.*

### Identification of Conserved Amino Acids in Subtilisin

**FIGURE 4:**
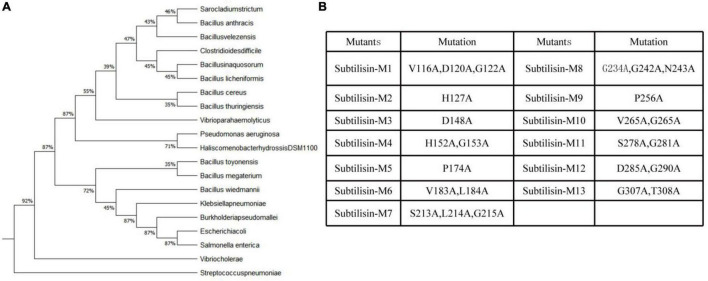
Multiple sequence alignment of subtilisins. **(A)** Phylogenetic analysis of 20 subtilisin amino acid sequences after grouping. **(B)** Identification of conserved residues in subtilisin for point mutation analysis. List of point mutation sites for subtilisin in each subtilisin construct. All selected conserved sites of amino acids were mutated to alanine. Taking M1 as an example, 116V, 120D, and 122G were mutated to A and are thus noted as V116A, D120A, and G122A.

Considering the domain conservation of subtilisin may be the result of evolution, multiple sequence alignments were performed to identify the conserved residues in subtilisin, which may be helpful to elucidate the mechanism for its immune functions. We conducted phylogenetic tree analysis (Figure 4A) and similarity analysis (Supplementary Figure 2) of 20 amino acid sequences of subtilisin. After comparing sequences with identities greater than 75%, a total of 13 conserved sites were selected. To determine which amino acids play a key role in subtilisin-induced immunity, we generated subtilisin mutants in which these conserved sites were replaced with alanine. In total, 13 subtilisin mutants were constructed (Figure 4B).

### Conserved Amino Acids of Subtilisin Were Crucial for Its Induced Resistance Activity

**FIGURE 5:**
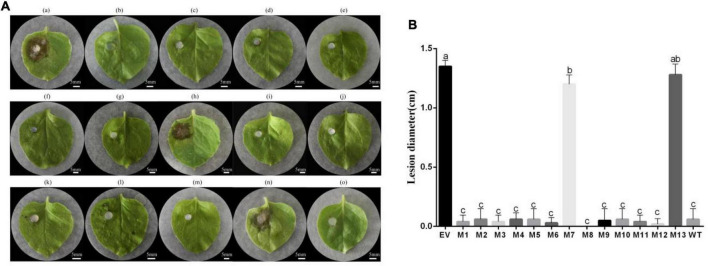
The transient expression vector of subtilisin mutants induced resistance in *N. benthamiana* against *B. cinerea*. **(A)** (a)–(o), respectively, are representative phenotypes of disease caused by *B. cinerea* in leaves of *N. benthamiana* expressing pK7WG2D control (EV), subtilisin-p1 mutants (M1), subtilisin-p2 mutants (M2), subtilisin-p3 mutants (M3), subtilisin-p4 mutants (M4), subtilisin-p5 mutants (M5), subtilisin-p6 mutants (M6), subtilisin-p7 mutants (M7), subtilisin-p8 mutants (M8), subtilisin-p9 mutants (M9), subtilisin-p10 mutants (M10), subtilisin-p11 mutants (M11), subtilisin-p12 mutants (M12), subtilisin-p13 mutants (M13), and wild-type subtilisin (WT). Photographs were taken at 3 days after inoculation with *B. cinerea*. Experiments were carried out with five leaves per treatment. **(B)** Lesion diameter caused by *B. cinerea* was measured in *N. benthamiana* leaves expressing EV, M1, M2, M3, M4, M5, M6, M7, M8, M9, M10, M11, M12, M13, and WT. Data presented in **(B)** are the means ± SD of lesion diameter of five leaves. Columns with different letters indicate significant differences according to Duncan’s multiple tests (*p* < 0.05).

To determine the induced resistance activity of the subtilisin mutants, systemic leaves of *N. benthamiana* expressing subtilisin and its mutants were inoculated with *B. cinerea*. The resistance of *N. benthamiana* expressing subtilisin-p7 mutants (M7, S213A/L214A/G215A) and subtilisin-p13 mutants (M13, G307A/T308A) to *B. cinerea* showed small differences compared with that of *N. benthamiana* expressing pK7WG2D control (EV) but lower than that in *N. benthamiana* expressing wild-type subtilisin (WT). The resistance of *N. benthamiana* expressing other 11 mutants except M7 and M13 to *B. cinerea* showed small differences compared with WT but higher than EV (Figure 5A). The lesion diameter of M7 and M13 showed significantly larger than WT but formed small differences compared with EV, and the lesion diameter of other 11 mutants except M7 and M13 showed significantly smaller than EV but formed small differences compared with WT (Figure 5B), indicating M7 and M13 reduced disease resistance to *B. cinerea* in *N. benthamiana*. Furthermore, the lesion diameter of M7 was smaller than that of M13.

**FIGURE 6:**
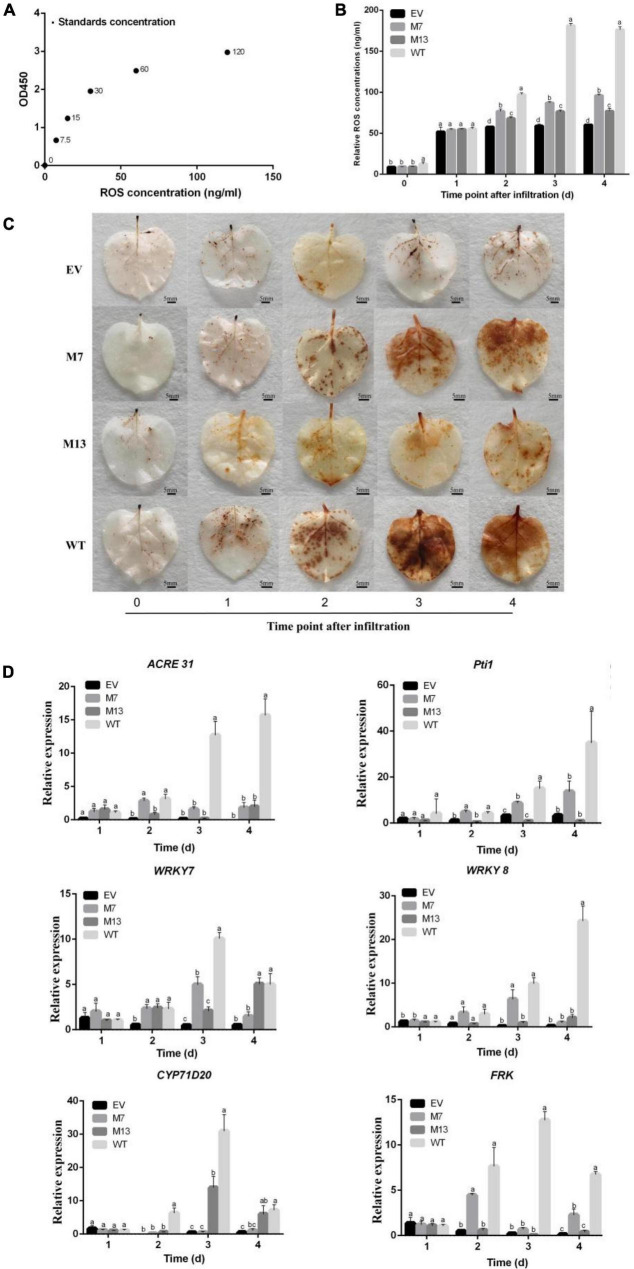
Changes in ROS and gene expression in response to subtilisin mutants. **(A)** The standard curve was generated from the average optical density OD_450_ obtained for each of the six plant ROS standard concentrations (0, 7.5, 15, 30, 60, and 220 ng/ml). **(B)** The ROS concentration of *N. benthamiana* expressing pK7WG2D control (EV), subtilisin-p7 mutants (M7), subtilisin-p13 mutants (M13), and wild-type subtilisin (WT) were measured by ELISA at various times (0, 1, 2, 3, and 4 days). **(C)** Accumulation of ROS was analyzed in *N. benthamiana* leaves expressing pK7WG2D control (EV), subtilisin-p7 mutants (M7), subtilisin-p13 mutants (M13), and wild-type subtilisin (WT) at 0–4 DAI. ROS was visualized by 3,3′ diaminobenzidine (DAB) staining methods. Brownish deposits were indicative of ROS. **(D)** Expression analysis of resistance-related genes *WRKY7/8, ACRE31, Pti1, CYP71D20*, and *FRK* in EV, M7, M13, and WT at 1–4 DAI. The samples were normalized against *Actin* and expression levels are represented as fold changes relative to the control. Data are means ± SD of three independent experiments. Columns with different letters indicate significant differences according to Duncan’s multiple tests (*p* < 0.05).

Reactive oxygen species plays a vital role in pathogen–plant interactions as signaling modules (Zhang et al., 2018) and has a crucial role in plants systemic resistance (Bóka and Orbán, 2007). Therefore, we generated the standard curve by plotting the average OD450 of ROS standard concentrations (Figure 6A) and detected the ROS accumulation and concentration in EV, M7, M13, and WT at different time points. Compared with WT, the ROS accumulation and concentration of M7 and M13 decreased at 2–4 DAI. The ROS accumulation and concentration of M7 and M13 were still higher than that of EV at 2–4 DAI. Furthermore, the concentration (Figure 6B) and ROS accumulation (Figure 6C) of M7 were higher than that of M13 at 2–4 DAI. These results established that the mutation of G307A/T308A and S213A/L214A/G215A impaired subtilisin-mediated ROS production in *N. benthamiana*, and the weakening effect of G307A/T308A was even greater.

To further illustrate the roles of G307/T308 and S213/L214/G215 in subtilisin-induced disease resistance, we analyzed the expression levels of the *WRKY7, WRKY8, ACRE31, Pti1, CYP71D20*, and *FRK* gene in EV, M7, M13, and WT. RT-qPCR analysis showed that *Pti1*, *ACRE31*, *FRK*, and *WRKY7* expression levels in M7 decreased significantly compared with those of WT at 3–4 DAI, among which *ACRE31* and *FRK* expression levels of M7 did not differ significantly from those of EV at 3 DAI, and *ACRE31, Pti1*, and *WRKY7* expression levels in M7 did not differ significantly from those of EV at 4 DAI. The expression levels of CYP71D20 in M7 decreased significantly compared with those of WT at 2–4 DAI. *WRKY8* expression levels of M7 were significantly decreased compared with those of WT at 4 DAI. *ACRE31, FRK*, and *Pti1* expression levels of M13 were significantly decreased compared with WT at 2–4 DAI. *CYP71D20* expression levels of M13 were significantly decreased compared with WT at 2 DAI and 3 DAI. *WRKY8* expression levels in M13 decreased significantly compared with those of WT, while not significantly different from EV in 3 DAI and 4 DAI (Figure 6D). *ACRE31, FRK*, and *Pti1* of M7 were significantly higher than those of M13 at 2 DAI. The expression levels of *pti1, WRKY7*, and *WRKY8* in M7 were significantly higher than those of M13 at 3 DAI.

## Discussion

In recent years, proteases related to plant immunity have gained increasing attention (Thomas and Van Der Hoorn, 2018). Subtilisin, a serine protease, is involved in plant–pathogen resistance and plays an important role in pathogen recognition and initiation of resistance-related signal pathways (Xu et al., 2020). Most of the subtilisin reported so far are directly or indirectly generated by plants in order to recognize one or more components secreted by the attackers and as a consequence activate defense responses to prevent pathogens invasion (Figueiredo et al., 2014). In this study, we observed a systemic effect of subtilisin in *N. bethamiana* against *B. cinerea*. The lesion diameter of the systemic leaf in NES was significantly smaller than that of CK at 3 DAI (Figure 1). Furthermore, subtilisin itself did not cause the inhibition of *B. cinerea in vitro* (Figure 2). On the basis of these results, we assumed that the induced protection is due to plant systemic resistance rather than directly bacteriostatic effect to the fungus. The result was in line with previous studies reporting that the protection exerted by AsES against *B. cinerea* (Hael-Conrad et al., 2015) was systemically induced. Establishment of systemic resistance was usually accompanied with potentiated activation of various cellular defense responses against other pathogens infection (Conrath et al., 2006). Thus, in an attempt to characterize the systemic resistance, the production and concentration of ROS were analyzed in a systemic leaves. NES showed a significantly higher ROS concentration (Figure 3B) and more brown DAB-stained precipitates (Figure 3C) than that of CK at 2–4 DAI. This implied that subtilisin could prime plants for an increased and faster capacity to activate ROS accumulation in systemic tissue, which was consistent with the results reported by Li on the PeFOC1 protein elicitor that can activate ROS burst and trigger systemic resistance in *N. tabacum* cv. *samsun NN* (Li S. et al., 2019). The same ROS burst was also found in Zhang’s research on SsCut protein elicitor (Zhang et al., 2014).

Pathogen-associated molecular pattern–triggered immunity (PTI) plays an important role in plant disease resistance against *B. cinerea* and was a potent elicitor to induce SAR. Therefore, we examined some typical PTI elements during the subtilisin-induced SAR to further investigate the relationship between SAR and PTI. WRKYs participated in mediating disease resistance and expression of resistance-related genes in SAR (Birkenbihl et al., 2012). *WRKY7/WRKY8* functioned downstream of MAPK during PTI. MAPKs were also reported to act upstream of ROS accumulation (Meng and Zhang, 2013). *Pti1* can induce ROS production in response to PAMP perception. *ACRE31* was rapidly induced by the elicitor and was involved in a later plant defense signaling cascade. Systemic signals were produced in the primary infected leaves and signal movement into distant organs (leaves). Tissues exhibiting SAR in the distant, pathogen-free parts of plants, displayed a “prepared” state associated with faster and stronger defence mechanisms (Ádám et al., 2018). The expression level of WRKY7 of untreated distal leaves in NES was significantly higher than that of CK at 2 DAI and 3 DAI. The *Pti1* expression level of NES was significantly increased compared with CK at 2–4 DAI (Figure 3D), suggesting that MAPK activation by subtilisin may be related to the ROS burst against pathogen infection. We also examined PTI marker genes, such as *FRK* and *CYP71D20* (Dagvadorj et al., 2017). qRT-PCR analysis showed that the expression level of *FRK* and *CYP71D20* was significantly increased in NES at 2–4 DAI (Figure 3D), implying that the generation of subtilisin-induced SAR may be associated with the ROS burst and the activation response of PTI. In line with previous results, it was shown that AsES induces a systemic protection against *Colletotrichum acutatum* in strawberry (Chalfoun et al., 2013). These results confirmed that there is a close association between subtilisin-induced SAR and activation of PTI.

Pathogen-associated molecular patterns have a certain degree of conservation across different classes of microbes to general microbial fitness, whereas effectors that either facilitate infection or trigger defense responses often exhibit species-, race- or strain-specific as a result of natural selection (Yang et al., 2017), and the common distinction between PAMP and effectors cannot always be strictly maintained (Thomma et al., 2011). Subtilisins are especially abundant in plants and have undergone evolution, and the subtilisin encoding gene sequence is highly specific. Subtilisin from *B. velezensis* LJ02 was evolutionarily closely related to *Sarocladium strictum* and *Bacillus anthracis* because it showed the highest similarity (Figure 4A). Conserved functional residues played critically important role in protein function. For example, Li demonstrated that conservative sites of the *Arabidopsis thaliana* natural resistance-associated macrophage protein, D72 and N75, were essential for transport activity (Li et al., 2018). Pawel Z suggested that Thr807, Thr812, Tyr815, and Tyr820 of receptor-like kinase ERECTA in the activation segment of the kinase domain were functionally important (Kosentka et al., 2017). Since several subtilisin homologs can all stimulate the defense responses of plants to *B. cinerea*, such as the fungal subtilase AsES (Ádám et al., 2018) and the subtilisin-like protease Bcser2 (Liu et al., 2020), we speculated that one or more conserved amino acids from the active site or adjacent residues may be essential for subtilisin to induced defense response in different species. Sites with more than 75% similarity of 20 homologous subtilisin sequences were selected, and a total of 13 conserved sites were obtained (Figure 4B). To identify conserved residues that may be important for the defense response induced by subtilisin, we generated 13 point mutants with alanine substitutions for those conserved sites and found that the lesion diameter of M1, M2, M3, M4, M5, M6, M8, M9, M10, M11, and M12 formed small differences compared with WT, but all M7 and M13 resulted in a significantly decreased resistance of *N. benthamiana* resistance to *B. cinerea* compared with that of WT by the *Agrobacterium* infiltration assay (Figure 5), indicating that G307/T308 and S213/L214/G215 were indispensable for the resistance induced by subtilisin to *B. cinerea*. The ROS concentration (Figure 6B) and accumulation (Figure 6C) of M7 and M13 showed decreased compared with WT at 2–4 DAI, and M13 exhibited lower ROS accumulation and concentration than M7. The expression levels of *Pti1, ACRE31, FRK, and WRKY7* in M7 were significantly decreased compared with those of WT at 3 DAI and 4 DAI. The expression levels of *CYP71D20* in M7 formed significantly decreased compared with WT at 2–4 DAI. *WRKY8* expression levels of M7 were decreased significantly compared to WT at 4 DAI. *Pti1, ACRE31*, and *FRK* expression levels of M13 formed significantly decreased compared with WT at 2–4 DAI, and showed no significantly different from that in EV. *CYP71D20* expression levels of M13 were significantly decreased compared with WT at 2 DAI and 3 DAI. Furthermore, *WRKY8* expression levels decreased significantly compared with those of WT and were not significantly different from those of EV at 3 DAI and 4 DAI (Figure 6D). These combined results suggested that mutations of G307/T308 or S213/L214/G215 directly affect the activation of plant defense responses. These amino acids may be the key sites for subtilisin to improve disease resistance. The reason why these amino acid sites activate plant defense responses, such as active oxygen bursts, is not known. This study provided theoretical guidance for further analysis of the key functional sites at which subtilisin can activate defense responses and contribute to the development of disease biocontrol strategies in plants.

## Data Availability Statement

The datasets presented in this study can be found in online repositories. The names of the repository/repositories and accession number(s) can be found in the article/Supplementary Material.

## Author Contributions

YW and ZL designed the study. JH performed the research, analyzed most of the data, and wrote the first draft of the manuscript. YY and RC contributed to refining the ideas and finalizing this manuscript. YW and ZL wrote the final draft of the manuscript. All authors contributed to the article and approved the submitted version.

## Conflict of interest

The authors declare that the research was conducted in the absence of any commercial or financial relationships that could be construed as a potential conflict of interest.

## Publisher’s note

All claims expressed in this article are solely those of the authors and do not necessarily represent those of their affiliated organizations, or those of the publisher, the editors and the reviewers. Any product that may be evaluated in this article, or claim that may be made by its manufacturer, is not guaranteed or endorsed by the publisher.

## Footnotes

1
www.ncbi.nlm.nih.gov/genbank/
