# Supplementary material for: Identification of Key Residues Essential for the Activation of Plant Immunity by Subtilisin From Bacillus velezensis LJ02
Source: Front Microbiol. 2022 Aug 15;13:869596. doi: 10.3389/fmicb.2022.869596 (PMC9421249; doi:10.3389/fmicb.2022.869596)
Supplement: Supplementary file 1 [file Data_Sheet_1.docx]

Supplementary Material

# Supplementary Data

**Supplemental Data 1. Related sequences of subtilisin**

**Sequence of subtilisin**

ATGGCGTTCGGCAGCACGTCTCCTGCCCAGGCGGCAGGGAAATCAAACGGGGAAAAGAAATACATTGTCGGATTTAAACAGACAATGAGCACGATGAGCGCCGCTAAGAAAAAAGATGTCATTTCTGAAAAAGGCGGGAAAGTGCAAAAGCAATTCAAATATGTAGACGCAGCTTCAGCTACATTAAATGAAAAAGCTGTAAAAGAGCTGAAAAAAGACCCAAGCGTCGCTTACGTTGAAGAAGATCACGTTGCAAAAGCGTACGCGCAGTCCGTGCCTTACGGCGTATCACAAATTAAAGCCCCTGCTCTGCACTCTCAAGGCTTCACTGGATCAAATGTGAAAGTAGCGGTTATCGACAGCGGTATCGATTCTTCTCATCCTGATTTAAAAGTAGCAGGCGGAGCCAGCATGGTTCCTTCTGAAACAAATCCTTTCCAAGACTACAACTCTCACGGAACTCACGTTGCCGGTACAGTTGCGGCTCTTAATAACTCAGTCGGTGTATTAGGCGTTGCGCCAAGCGCATCTCTTTACGCTGTAAAAGTTCTCGGCGCTGACGGTTCCGGCCAGTACAGCTGGATCATTAACGGAATTGAGTGGGCGATCGCAAACAATATGGACGTTATTAACATGAGCCTCGGCGGACCTTCTGGTTCTGCAGCGTTAAAAGCGGCAGTTGACAAAGCCGTTGCTTCCGGCGTCGTAGTCGTAGCGGCAGCCGGTAACGAAGGCACTTCCGGCGGCTCAAGCACAGTGGGCTACCCTGGTAAATACCCTTCTGTCATTGCGGTAGGCGCTGTTAACAGCAGCAACCAAAGAGCATCTTTCTCAAGCGTAGGTTCTGAGCTTGATGTCATGGCACCAGGCGTCTCTATCCAAAGCACGCTTCCTGGAAACAAATACGGCGCGTACAATGGTACGTCAATGGCATCTCCGCACGTTGCCGGAGCGGCTGCTTTGATTCTTTCTAAGCACCCGAACTGGACAAACACTCAAGTCCGCAGCAGTTTAGAAAACACCACTACAAAACTTGGTGATGCTTTCTACTACGGAAAAGGGCTGATCAACGTACAGGCGGCAGCTCAGTAA

**Supplemental Data 2. Amino acid sequence alignment, classification, and phylogenetic tree analysis**

**Supplemental Data 2-1 : Subtilisin amino acid sequences**

>Bacillus velezensis

MAFGSTSPAQ AAGKSNGEKK YIVGFKQTMS TMSAAKKKDV ISEKGGKVQK QFKYVDAASA

TLNEKAVKEL KKDPSVAYVE EDHVAKAYAQ SVPYGVSQIK APALHSQGFT GSNVKVAVID

SGIDSSHPDL KVAGGASMVP SETNPFQDYN SHGTHVAGTV AALNNSVGVL GVAPSASLYA

VKVLGADGSG QYSWIINGIE WAIANNMDVI NMSLGGPSGS AALKAAVDKA VASGVVVVAA

AGNEGTSGGS STVGYPGKYP SVIAVGAVNS SNQRASFSSV GSELDVMAPG VSIQSTLPGN

KYGAYNGTSM ASPHVAGAAA LILSKHPNWT NTQVRSSLEN TTTKLGDAFY YGKGLINVQA

AAQ

>Sarocladium strictum

MRLSLVLALL PVAFGAPTRR DEPAPLHVPR DVDSLIKDTY IVKYKDITAF SAVDEGLKLL SGKPKHIYKG AFKGFSGKID AKTLELLRDD PSVDFIEQDA IVTLAAYTTQ ASAPWGLARI

STRQRGPTGY TYDDSAGAGT CSYIIDTGIQ ANHPNFGGRA FQLVSYQGSN ADGNGHGTHV

AGTIGSTTYG VAKRTTLLGV KVLSDSGSGS TSGIIAGINY VVSDSRSRSC PNGSVANMSL

GGGYSASLNS AAKSLIDNNI FLAVAAGNEN QNAANVSPAS EPTVCTVGAT TSADAKASFS

NYGSGVDIFA PGQSILSTWI GSSTNTISGT SMASPHIAGL AAYLAGLEGF PGAQALCNRI

VALATTGVIT GLPSGTPNRL AFNGNPSG

>Clostridioides difficile

MRGMRIRGAG LLGSALVAAL IATSASPAAA VNPPRIDPGA LIRSAPVAPP EPTEQRHLCN

TTELTRNVSK STAAQSMMNL PEAWRFSRGQ GQRVAVIDTG VTPHPRLGRV VAGGDYVSGG

SGLDDCDAHG TLVAGIIAAR PSTADAFAGV APAASIIAIR QSSSAFQKKD DRARDDAAPS

VGSGYGSVRT LAHAIVRAVD LRATVINISE VACAPASDSV DDRALGAAIR HAYDRDVVVI

VAAGNLTRDG ACQNQNPAPA ADDPGGWRSV STIASPAWYS PYVLTVGSVD ADTGSPSDFS

LHGPWVSVAA PGTDIVSLDS RRGSRRLASA QESDGGPIPL IGTSFAAPYV AGTAALIRSR

FPELSAREVM ERIIATAHSP GDGHDQRIGH GVVDPVAALT AELPTDQRPD RASAPIAAPP

VAQPPDHSAR TVAIVGSAVA AIVIAAVLAI ALPHRRVRKL DPDEF

>Streptococcus pneumoniae

IIEVFEEHFD RAITCEMFSD SSTFASINFS EYGISKSKFQ QYLRDSCFIE NFGVEHTTVS

DIQNSIVTFY DVHTDIFRLL NKLNIDISEA NIMNQTTVLL DEKNIELLLS KAPYLVSMIV

EDFSKLSVDD FSLDNNDLKI NLPSPMNEPV VGVIDTLFDK RVYFNEWVEY HDFVSPDISK

DSQDYKHGTA VTSLIVDGAN LNPNLDDGCG NFRVRHFGVS LQSGFNSFTI IKQIKEIVSQ

NADIKVWNLS LGSNDEIREN FISAEGALLD EIQFENDVIF IIAGTNASVI NGKRKRIGAP

ADSLNSIIVN SVDFNNQSVS YSREGIVLSF FVKPDVSYYG GGNGDFINVC EPLGLGRVAG

TSFAAPFIAR KMAYLIHIMG LSREEAKALL IDAAIPWNDK KTFTDLSLIG NGIVPIKMDD

ILSTPDDEIK FIVSDISRAY DTYNYDFPVP ISSESYPYVA KATMCYFPNC SRKQGVDYTN

TEMQLTFGRL KSDGIKSINK DNQHAEDTPG YVREKSARNI FRKWDNVKHL GEKFSSRKKA

KPILNPSNPQ WGMSVKTIER LRNDDGYGIR FGVVVTLKEL NGVNRIEDFI QQAELRGWLV

NRLQVEAQVD LFNSLNEEIE FE

>Escherichia coli

MAKTNFLIGR GELLTSDIPG PKRVPGKAEV YSLYESKQRL LPQIASTIEN FDDLPSQACP

YDYAVAKLTL NPSYIARSFF PAKMLKSTGL TSIGSRTVKI TPQSWKRKVT VSEVTTTQMF

VAGKRESFKE LPHWVSGLKE FSDEAIDFAR IERFDAYLPE ERIITIGEQD SSYFEVGVHL

LPDNEQDLVQ QQFVQFASNL NIKVHTSLAF TAGTLWFVPI QADRITISKL AKFTFVRVIR

PVPRLRGIRP IQRNYGPLAQ CKLPAEGPVS SEIKVAILDG GLPSEHAISP WLKSYRVLDD

HAEDDPDGLE HGLAVSSAFL FGPISNKSEA KRPYSYIDNL RVLDNKTCQE DPLELYRTLG

FIEEVLLSRQ YQFINLSLGP DLPIEDTEVH AWTSVIDDLL SDGETLMTVA VGNNGEMDRK

SGNARIQVPA DCVNALAVGA CDTVNDTEWK RAPYSAIGPG RSPGVMKPDL VAFGGDTDEY

FHVLGKGKKP VIIPQLGTSF ASPYALRAAV GVRAILGNDL SLLAIKALLI HASKQLDHPC

TEVGWGKLPE DLNDIIVSPD GVARIVYQGE LKPGKYLRAP LPIPEGGLKG RINLAATFCY

ATTTDPQDPA SYTKAGLEVT FRPNDSKIKD GKQNAETKGF FELAKYSTES ERRSDMGKWE

TVLHNSKNML GSTLKNPVFD IHYNAREAGA SIASHKAEKI KYALIITIKA SKHQDLYNEI

LRTYNQVLVP IQPQTSIPIR S

>Salmonella enterica

MAKTNFLIGR GELLTNDIPG PKRAPGKAEV YSLYESKQRL LPQIASTIEN FAKLPSRACP

DDYAVAKVTI NPSYIARSFF PAKMLKSTGL TSIGSRTVRV TPQSWKRKVA VSEVTTTQMF

IAGKRKSFEE LPHWVSGLKE LTDESIDFAR IEKFEAYLPE ERIVTAGDMD SSYFEVGVHL

LAEDGQDLIQ QQFVRFASTL NIKVHTSLAF TAGTLWFVPI QADRIAIDKL AKFTFVRVIR

PVPKLRGIRP IQRASGPLAQ CKLPAEGPVS SEIKVAILDG GLPSEHAISP WLKSYRVLDD

HAEDDPDGLA HGLAVSSAFL FGPINNKSEV QRPYSYIDNL RVLDNKTCQE DPLELYRTLG

FIEEVLLSRQ YQFINLSLGP DLPIEDTEVH AWTSVIDDLL SDGETLMTVA VGNNGEMDRE

SGNARIQVPS DCVNALAVGA CDTVNETEWK RAPYSAIGPG RSPGVMKPDL VAFGGDIDEY

FHVLGKGKKP VITPQRGTSF ASPYALRAAV GVRAILGSDL SQLAIKALLI HASKPLDHAC

AEVGWGKLPE DLNDIIVSPD GVARIVYQGE LKPGKYLRAS LPIPEGGLKG KINLTATFCY

ATTTDPQDSA SYTKAGLEVT FRPNDSRVKD GKQNAETKGF FELAKYSTES ERRSDMGKWE

TVLHSSKNML GSTLKNPVFD IHYNAREAGA SIASHKAEKI KYALIITVKA AKHQDLYNEI

LRAYNQVLVP IQPQTSIPIR S

>[Bacillus cereus](https://www.ncbi.nlm.nih.gov/protein" \o " (firmicutes))

MLFLYLDYIV LITMLTLKKC RKEVNKVKKI LILILTGLVL CSSLFYFLFY SESTLSKTKS

NIPYKTKKQS VTWGRKAVIE DKFTHIQNTI KVAILDSGIY KEHEDLKGKV VKEFNTINLN

TPITDETGHG TAIAGIIAAE NNNLGILGVV PNVLLYDVTV LNNQGKGDVD HLIKGIRWCI

EQQVNILNIS FGLQSESPEL KKAIDEAVDS GIIVVAASGN TYGLSVDYPA KYENVISISS

VNKEFKRASS SAKGKIDFAA PGVDILSTNQ DGGYSIYSGT SFATAFATGV ISVLLAESDN

KLNLDQIKKT LIERSIDLGS KGYDNEFGHG LIQK

>Pseudomonas aeruginosa

MAYEHLRLER EAPSTERHPR RHPGIRPPAD PRAHGAALAG RLDQARQRAM AEDVGGFDDR

KLLKIRLRAG DKSVPAFDAI PGVEIVSQED ESIVLAFATD DGLSEFESRL ATLARDGVVT

RKELFYVIED FDHWTPQDRT GAALLEQGFP AAPTFMLDVE LWPQERQDKR QQMVRAFLDW

LHAQGIERLD DIQQPSLVMV RVRCNGAQAE QILHHRDVRT ADLPPRLGVA VQLLHTDINQ

FPPIDPPSDD APSIAVLDSG LTRGHSLLGA AVGDAQGYLA PHRSADDTDP HWHGTFVGGL

ALYGDVHSAI QQGQFVPQLR LFSGKVFEDD GQDQTEFVEK AVEEAVRELH AQYGCRVFNL

SYGDLNKVYD GRHVRGLAYT LDRLTRELGV LFVVPAGNLL SSQLPADTRA SYPDYLFEGH

ARLLDPATSL NALTVGGLSL NEATRNAQRH PNTIEDHVLA RAEQPFPLTR SGPSASGAIK

PDVVAHAGNI ALRRTGGGTD HAGLGVVSLN GGFALGPAFK EDIGTSYAAP QVAHQAARLL

AEVPDASPSL LRALIGAHAR WPQACEALLN PGNNAEGRDK LLRLVGYGRV DDAALFRSLD

HTVTLLAEER VGNDQHHFFE LPLPDSFWDG GRRTREVTVA LAYSPAVRTT RLDYRRAKLW

FHLVTAGSLD EITQAYRRNR EEGMGERANG RWLPNDTRKN GTLQVSRWRF KQALANGHKV

FVVVTRQDSL WSDGRDGDEP YAVAVVLADR EQANAQLYAQ VQAALQARAQ ARARARIGGR

A

>[Bacillus thuringiensis](https://www.ncbi.nlm.nih.gov/protein" \o " (firmicutes))

MEFKIQSNVI EIQSASPIQV VDWGVNVVQA PEMWSITKGE GIKVAVLDTG IDATHPDLAS

NYKKGMNFTT SNFTDIMDRQ GHGTHCAGII AGCDNSIGIV GVAPKAELYI AKVLVDDGSG

SVEAIVKGID WAISEQVDII SMSLGSSADP GPVLHNAIKR AHEAGIIIVA ATGNENTHVG

WPASYDEVIA VGAINQNLDR ANFSNFGSET DIAAPGVDIY STYPVGRYAK LSGTSMATPM

VAGVIALILA RYRDIGKKLT PDQIMQLIRE HSVDLGQKGT DDMFGNGLVS IHELMKTS

>Bacillus inaquosorum

MRSKKLWISL LFALTLIFTM AFSNMSAQAA GKSSTEKKYI VGFKQTMSAM SSAKKKDVIS

EKGGKVQKQF KYVNAAAATL DEKAVKELKK DPSVAYVEED HIAHEYAQSV PYGISQIKAP

ALHSQGYTGS NVKVAVIDSG IDSSHPDLNV RGGASFVPSE TNPYQDGSSH GTHVAGTIAA

LNNSIGVLGV APSASLYAVK VLDSTGSGQY SWIINGIEWA ISNNMDVINM SLGGPSGSTA

LKTVVDKAVS SGIVVAAAAG NEGSSGSSST VGYPAKYPST IAVGAVNSSN QRASFSSAGS

ELDVMAPGVS IQSTLPGGTY GAYNGTSMAT PHVAGAAALI LSKHPTWTNA QVRDRLESTA

TYLGNSFYYG KGLINVQAAA Q

>Vibrio parahaemolyticus

MAKNKNYLIG RAEVLASLTP APKMKFDRTP LYSISESKAR LEPQLKNVCD ELSKMPSELC

PNDLSVTRMT LHPSFIAKGH FPKKFLRQMG VQSIGSTSSN VKPDRWTRVG EPEKSPSTSL

YLSGKRQDIL EFSQKLSQYN DESPAAEDLE KIWSIEVIDA ESKIKKGLPQ SVQGYFEVGL

QLLPGHHSST IKKEFVKLAN KYEYECRVDL GFDVSNLWFL PIIGKESHLK ELAKFSFVRV

VRPVPAMRSF RPIVRANSLG SGVNLPKEPP YAADVKVAIL DGGLPKLSAV QPWVQNYRLS

NEEAADLDGG PDHGLGVTSA FLFGPLDPED TVPRPFSYVD HHRVLDADIH GDDPLELYKT

LGHIEEILLS HQYEFINLSL GPDLPIDDDE IHPWTSLLDT YLADGRTFLT IAAGNNGDND

STLGLDRIQV PSDCVNAISV GAASTQCPSW QKTIYSAIGP GRAPGRVKPD LVAFGGSPKE

YFHVVSSDIP PRAVPQCGTS FSAPYLLRKA VGVRALMGHE ISPLAIKALL INNANSNGYV

QEHVGWGKVP KSIETLIESP DGEAKIIYQG ELSPGKYLKV PVPIPDTGVD GKVTISATCC

YSTPVDPQDT SMYTRAGVEI SWSVNGKNEQ FFKQKKIASE AELRADAAKW ESVLHAEKTK

IGSKMGEPTF EIHYMAREGG APVSSSRAET IKYAFVVTLK APKHKSIFND ILDTYADILT

EIEPRITTDV TVEV

>Klebsiella pneumoniae

MATNFLIGRG ELLASAVVGP RRGMDKSEVY TFAEAKQRLI PQVEQATSDL DDLPAVACPN

DFAVATLTLN PSYIARSFFP DTLLRENNLQ SIGSKTVKIK PDGWKRKAEV TEFPTTQIFV

AGKRSSFKNI IPWVQSLDDG SDEAIDFARI EMFSAYKPEE RIVPFSNREA GEFFEVGIHL

LPSNNSDLVQ SAFMGYAEDN GITLYSELSF TAGNLWFVPV QCGQEKIETL SLFTFVRVIR

PVPPLRGIRP IQRASGVKIQ TLLPSDPPVS DLRVAILDGG LPKQHSIQKW LNSYRVMDDG

ASDDSDALQH GLAVTSAFLF GPLKARTMAP RPYSYVDHLR VLDADICNEH PLELYRTLGL

IEEVLLSRQY QFINLSLGPN LPIEDSEVHA WTSVIDDLLS DGETLMTIAV GNNGEMDKES

GNARIQVPAD SVNGLSIGAA NSTDSNKWAR SSYSAMGPGR SPGVIKPDLV TFGGEEREYF

HVLTDDTNPV IAPQMGTSFA APYALRTAVG IKALLGGDLT TLAIKALMIH SCSQKDYSHA

EVGWGKLPEN INDIIVSPNG VARIVYQGEL KPGKYLRAAL PIPDGGLNGM VNLTATFCFS

TSVDPQDSAS YTKAGLEISF RPDESKKKQG AQNADTKGFF ELKKYANEQE RRSDMGKWET

VLHASKSMRG TSLNKPVFDI HYNARQGGGA IQGNKAEKIK YALILTITAN KHPDLYNDIL

RSYNQILAPI QPQATIPVRT

>Vibrio cholerae

MVSKRKHLDV ARFFIDEPFK SKRPGRNSGV PGRDRNQHGS YLAGLYQNLI NAYEQKRKQQ

INPITDDSGI YVEIIGVDGC KLPLDSLDNR DFKLCSCQMR GDREFALIFI PEDKRDTFLK

KIQQYLDPQK DGKPNKEGVS FPRNHTLIDS ISEIRLANLE SFWTDPIDLF PADRNLDVWW

ELWLKSNTVD DVKKIAESLA ERVDGRLGNT SLSFFNSFVV LIKASVNKLE KAPELISNLE

EIRKAKETPV PIISSSPKEQ QEWLKSISDR VSLSDNITTS VSILDTGINY NNMLLSKVCC

DDFSVSWDPD WPKYDQYQAL APFNEHGSLQ AGLAAFGNLM DVVLGNSAIQ LSHVIESARI

LPPQGNNDPF LYGAITVGTA SKLEVDRPDI NRVYSLAVTS DHERESGRPS SWSAEIDQFT

SGMQDGKRRL FVISAGNNLD IRPDQDYWDQ VNLAQIEDPA QAWNAITVGA YTEMTTNDDP

YFEGWSPFAM AGDVAPSSRS SVNWAWRKQA PFKPDVVAEG GNRLLSPDKT ELSNEDTVGL

LTTSGKTTGQ VFERGSDTSA ACALVSRCAA QLTAEYPELW PETIRGLIIH SAEWTPRMME

RFGLLSAVHS PKVAKETLLR TVGYGVTNID RARYSADHAL TLIAEGEIQP FIKSQDANAS

SDPKLNQMKL YQLPWPLAEL QNLPPELEVK LKVTLSYFIE PNPGRRGYRT RYSYQSHGLR

FETIRPGQSI ANFRSYINGL ANMDDYDGPE GDNDGWFLGS QLRTRGSIHS DHWTGSAQDL

ADMHTIAIFP VGGWWKYKTA EERWENRVRF SLLISIEVPD ESVDIYSVIE NQIEVAIENQ

VAIEIVT

>Haliscomeno bacterhydrossis

MFIRLLQGFT KGHNHNPMKH ILFNALCLFG IVLSSGSVVF AQKIEREVLE AFSTQGHTEA

LVVLVEQADL RFAQQLPNKE AKGEYVFQKL KRTAERSQAP LWNILHSAEV SATPLWIVNA

LYIPRLDKAT ASLLAAHPAV EAIVSNPSTY FSAPRREIEN SSLRSALEWG IEKIGATAVW

RKGFTGQGVV IAGQDTGYDW EHITLKQKYR GIEGSSINHN YNWHDAINKG SPLNADTLNP

CGFSSKIPCD DGSHGTHTMG TMLGDDGAGN QIGVAPGARW MGCRNMDRGW GSPFTYLDCF

QWFLAPTDLK NQNPNPKLAP HVINNSWGCP EIEGCGPGNW GLMEKAIINL RAAGVVVVVS

AGNDGSQGCG SVNDAPAFFP QSFAVGATRI DDVIARFSSR GPARWGDSLV LKPNVSAPGQ

GVRSAVPGGF AVFSGTSMAG PHVAGAVALI ISANPALAGQ VEAIESLLEK TAVPLIDSMS

CSGLRGNISP NAVYGYGRIN VLAAVEAALL ITPVDTPTQQ KIAIQVAPNP VIEGTLQVEL

GALPDAGRLE VLDPTGRLLQ RHVLPQANFQ TKTLTLGTLP AGTYFIRVIA GNVYGYQKIV

KI

>Bacillus licheniformis

MMRKKSFWLG MLTALMLVFT MAFSDSASAA QPAKNVEKDY IVGFKSGVKT ASVKKDIIKE

SGGKVDKQFR IINAAKAKLD KEALKEVKND PDVAYVEEDH VAHALAQTVP YGIPLIKADK

VQAQGFKGAN VKVAVLDTGI QASHPDLNVV GGASFVAGEA YNTDGNGHGT HVAGTVAALD

NTTGVLGVAP SVSLYAVKVL NSSGSGSYSG IVSGIEWATT NGMDVINMSL GGASGSTAMK

QAVDNAYARG VVVVAAAGNS GSSGNTNTIG YPAKYDSVIA VGAVDSNSNR ASFSSVGAEL

EVMAPGAGVY STYPTNTYAT LNGTSMASPH VAGAAALILS KHPNLSASQV RNRLSSTATY

LGSSFYYGKG LINVEAAAQ

>[Bacillus wiedmannii](https://www.ncbi.nlm.nih.gov/protein" \o " (firmicutes))

MKKITIHNLP LFAFTIFSLL IFFIGGLNEK NVFAAEHDIK KDYLIMFNNT VDEQLIVEHG

GVIKETFENL PIIKAEFSYN PEKVLKHNSN VSEVEVDQTI KALGDSNTQT NPSMTQSKSI

NTIPFTGKGV SVAILDSGID TEHTDLKIKD GISFVENHPS FDDDNGHGTH LAGIVAAQDN

ELGMTGIAPN VDLYAVKVLD KYTNGKYSTV VKGIDWAIEH NVNIVLMSLG GKKESAFFKE

AMDKAYQKGI LLIASAGNEG YKDGNTISYP AKFQSVVAVG ALDKNDARGF LSSKGDELEL

MAPGVDILST WKDGNYRLDS GTSMAAAHVA GVASLIFEKN PYLSNKQVRE IMNKAAIPLG

NAFEYGNGKI NVDYALNMAD

>[Bacillus toyonensis](https://www.ncbi.nlm.nih.gov/protein" \o " (firmicutes))

MKMFQRIGVS AVIVSTFLGI GDNVNIVTAD SIEQQSKLGI EQETDKQIIV KFSADLKLPY

EDGIEKQIQS ETNDKVLEDF LAEYPDVTFT RLFTSVSPEE IQNLEVKAPN NVSTSLLNYY

ILQTKNDGRE ETLVDKLKAS SLIEDVYMKK QEKIIPPEVK QLSVSLTPNN NPRFNKQGYL

EAAPYGINAP FAWGVQGGNG NDVTFVDMEY GWLLNHEDLV HRNIELMSGR NIDQHVGHGT

SVLGIVSSED NEVGNIGIAP KAKAKVISQI RDNGQYNTAD AILSAVNHLE AGDVLLLEAQ

ASFDGYGDKY LPVEVQPDIF DAIRAGTDKG VVIIEAGANG WNDLDQFKDR NGKQILNRNS

KDFKDSGAIM VGAGSSSFPH ERMWFSNYGS RIDVYGWGEN VDTTTAEQSR SAVNLYTSSF

SGTSSASPII AGAATLVQSI AKENVGQPYR PSELRTILSN HNTGTKSKDP YSDKIGILPD

LKSILVNLGY ERRELNGGNE LQVTENEPNN EPKQANKINF HTPMKGTLHN RDNVDVFTFQ

IDSPETINIS LLNEKNIGMT WVLHHESDVN NYVAYGENEG NVVKGTYNAK PGKYYLYVYK

YENKDGSYVL NIK

>Bacillus megaterium

MLQRLLLSFL ILFLLVSCDN QSIEVSFSQT DCKKNWSLCP LFPNNTRISM PTEPVKVAIL

DSGIDNSHPL LKEKIVKSFD ARRETNITKD KFGHGTAIAG IIGASSNKQT IQGISPNVEI

YDVKVLNDKG GGEIKDVVNG IEWSIKQNVD ILNLSFGFQK DDPTLKQAID YAVDNNIIVV

AAAGNTLGFS ADYPAKYNNV ISVSSVNKQM ERDKFAAKGK IDFVAPGVDI PVLTPTKKII

TVSGTSFSAA YVSGIIANIL AKKPNVQNFD AVLTELKTSS KDLGEKGLDN EYGNGLVQFK

>[Bacillus anthracis](https://www.ncbi.nlm.nih.gov/protein" \o " (firmicutes))

MKNKIIVFLS VLSFIIGGFF FNTNTSSAET SSTDYVPNQL IVKFKQNASL SNVQSFHKSV

GATVLSKDDK LGFEVVQFSK GTVKEKIKSY KNNPDVEYAE PNYYVHSFWT PNDPYYKNQY

GLQKIQAPLA WDSQRSDSSV KVAIIDTGVQ GSHPDLSSKV IYGHDYVDND NVSDDGNGHG

THCAGITGAL TNNSVGIAGV APHTSIYAVR VLDNQGSGTL DAVAQGIREA ADSGAKVISL

SLGAPNGGTA LQQAVQYAWN KGSVIVAAAG NAGNTKANYP AYYSEVIAVA STDQSDRKSS

FSTYGSWVDV AAPGSNIYST YKGSTYQSLS GTSMATPHVA GVAALLANQG YSNTQIRQII

ESTSDKISGT GTYWKNGRVN AYKAVQYAKQ LQENKAS

>Burkholderia pseudomallei

MSQTNFLIGR GELLTHDIVG PRRMPGKAEV YTFAQARERL TPQFRSAAKA LDQLSTDACP

GDFAVARLMM NPSFIARSYF PTALLRSTGL ESIGSRTVKV KPQAWTRKGQ PQETTTTELF

VAGKRQAFRN LSQWTETIDA ESDEGDDLAH IERFAAFAPA DRIVSLGGPK DRFFEVGLHL

LPDEDPALIQ KAFVKFAQRD GVKVHSEVDF VAGNLWFVPV EGKHREVERL ASFAFVRVIR

PVPKLRGIRP LQRSGGPTVA CSLPTEQPLS SEPRVAVLDG GLPKHHPIGP WLGSYRKLDE

DADDDPDGPE HGLGVTSAVL FGPIHPNGTA GRPFAPVDHL RVLDQESAGE EPLELYRTLG

LIEQVLLSRS YEFINLSLGP DLEVEDQEVH AWTSVIDELL SDGDTLMTVA VGNNGERDRE

LGYSRVQVPS DCVNALAVGA ADDTGADWAR APYSAIGPGR SPGVVKPDLM AFGGNPASKY

FHVLAPNAKP VLTPQLGTSF AAPYLLRSAV GIRAILGGNL TPLAIKALLV HAADPGEHDP

IEVGWGKIPE DTLDIITCPD GVARVVYQGE LKPSKYLRAS LPLPKEGLTG NVRLKATFCY

ASPTDPQDAA AYTKAGLEVV FRPNDEKIKD GKSNADTKGF FDLKKFATEQ ERRSDQGKWE

TVLHGAKTFR GSNLKNPVFD IHYNARTGGG RATGAEKIRY ALILSVEAPK HADLYNDILR

AYAKTLVPIQ PQVSLPIRVR

# Supplementary Figures and Tables

## Supplementary Figures


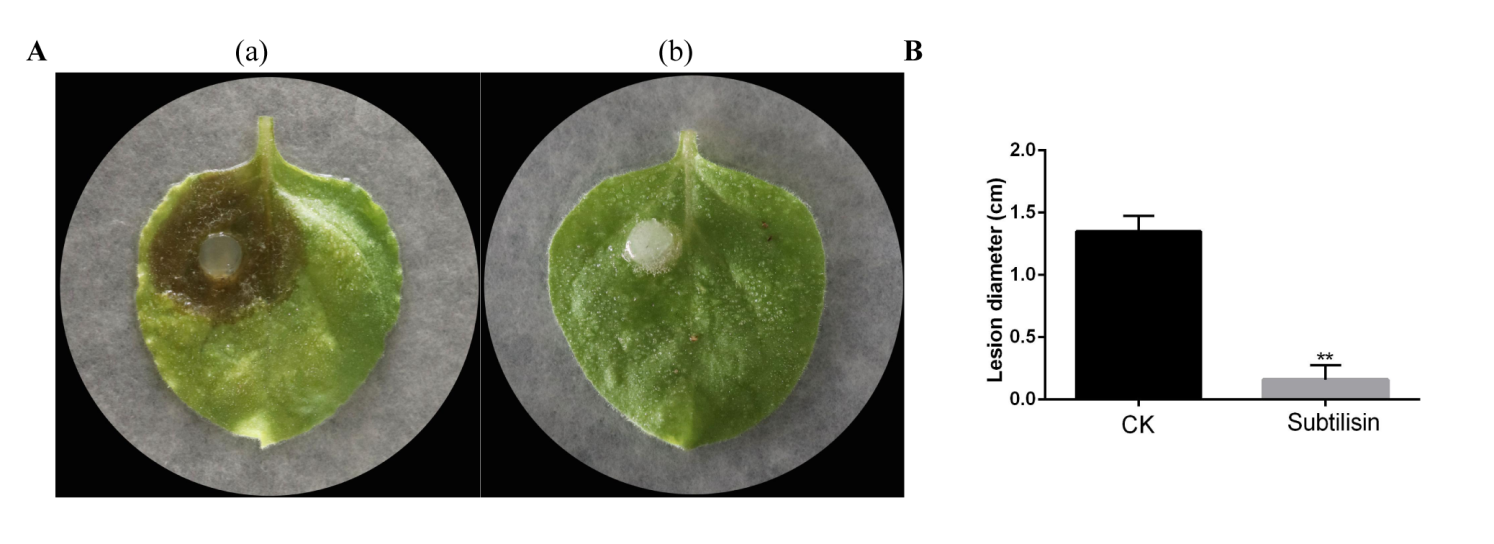


**Supplementary Figure 1.** Subtilisin induced resistance in *N.benthamiana* against *B.cinerea.* (**A**) Representative phenotypes of the disease caused by *B.cinerea* in *N.benthamiana* leaves infiltrated with buffer (a) and 100 μg·ml^-1^ subtilisin protein (b). Photographs were taken at 3 days after inoculation with *B. cinerea.* Experiments were carried out with five leaves per treatment. (**B**) Lesion diameter of disease caused by *B.cinerea* in *N.benthamiana* leaves infiltrated with buffer (CK) and subtilisin protein (Subtilisin). Data are means ± SD of lesion diameter of five leaves. Columns with different letters indicate significant differences according to Students’ *t*-test (** p < 0.01).
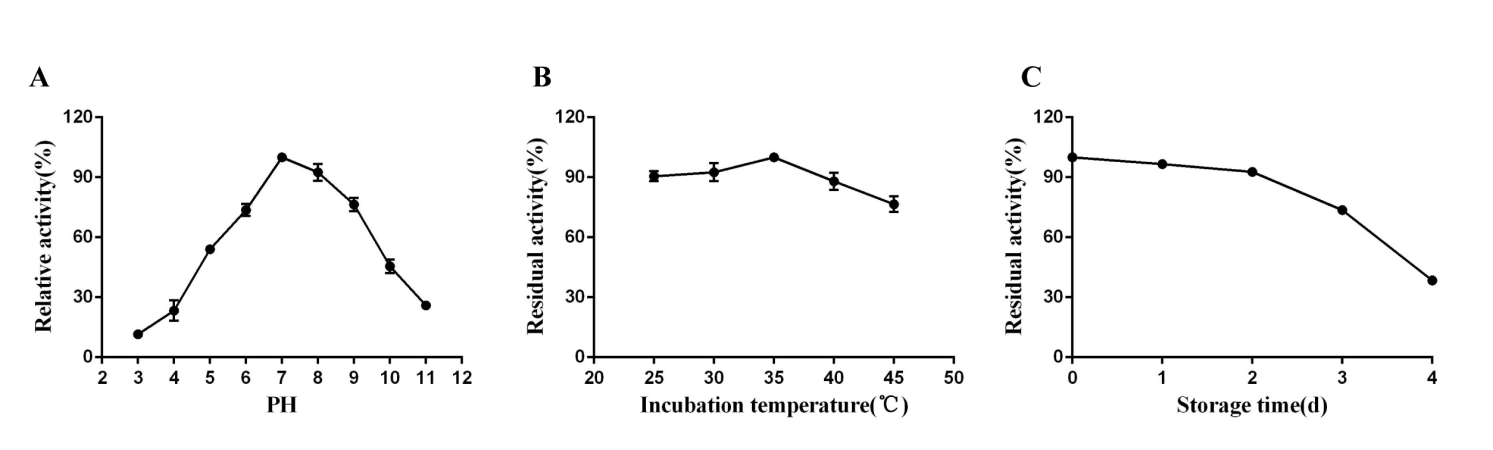


**Supplementary Figure 2.** Effects of treatments or conditions on the activity of subtilisin protein measured using the chromogenic substrate N-succinyl-Ala-Ala-Pro-Phe-pNa. (**A**) The pH dependence of the reaction was studied using the following buffers: citrate (pH 3.0–5.0), Tris–HCl (pH 6.0–8.0), and carbonate (pH 9.0–11.0). (**B**) Aliquots of the enzyme were pre-incubated in 50 mM Tris–HCl buffer (pH 6.0) for 30 min at 25, 30, 35, 40 and 45 ℃ and immediately assayed for enzymatic activity. (**C**) Aliquots of the enzyme were incubated with the substrate at pH 6.0 for 30 min after a preincubation of the protease solution in the incubator (28 °C) for 0, 1, 2, 3 and 4 d. Data are means ± SD of three independent experiments.


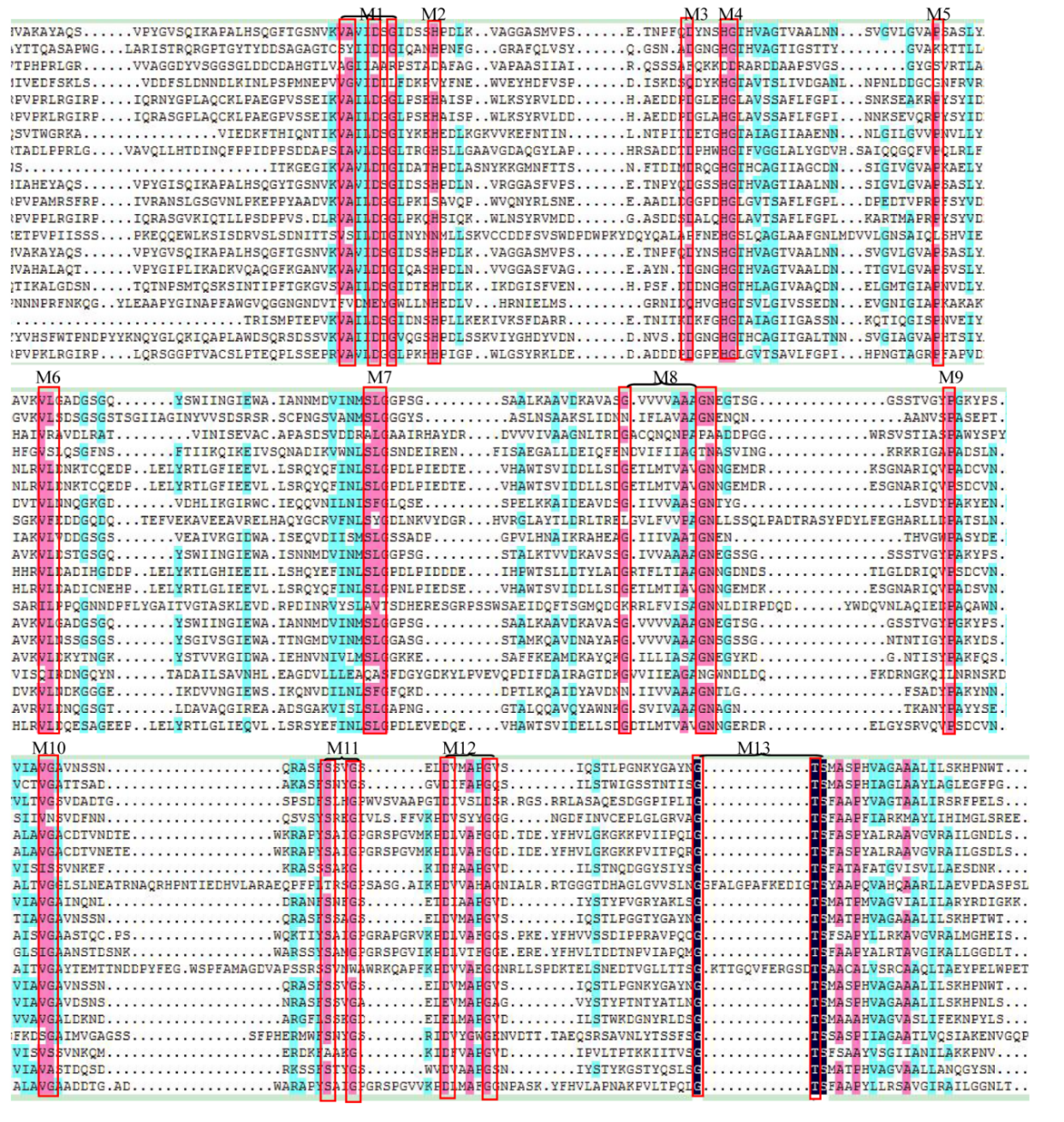


**Supplementary Figure 3.** Alignment of the conserved residues of subtilisin for point mutation analysis. Conserved sites (M1-M13) marked in the figure were selected for mutation.

## Supplementary Tables

**Supplemental Table 1：Subtilisin cloning primers**

| Primer ID | Sequence |
| --- | --- |
| Subtilisin-F  Subtilisin-R | GGACAGCAAATGGGTCGCGATGGCGTTCGGCAGCACGT  GGTGGTGGTGGTGGTGCTCGATTACTGAGCTGCCGC |

**Supplemental Table 2：Subtilisin prokaryotic expression vector construction primers**

| Primer ID | Sequence |
| --- | --- |
| pET-28a-Subtilisin-F  pET-28a-Subtilisin-R | GGACAGCAAATGGGTCGCGATGGCGTTCGGCAGCACGT  GGTGGTGGTGGTGGTGCTCGATTACTGAGCTGCCGC |

**Supplemental Table 3：Subtilisin transient expression vector construction primers**

| Primer ID | Sequence |
| --- | --- |
| PK7WG2D-subtilisin-F  PK7WG2D-subtilisin-R | attacgccgaggcctATGGCGTTCGGCAGCACG  atagggaagaggcctTTACTGAGCTGCCGCCTGTAC |

**Supplemental Table 4：Subtilisin mutant vector construction primers**

| Primer ID | Sequence |
| --- | --- |
| PK7WG2D-subtilisin M1-F  PK7WG2D-subtilisin M1-R  PK7WG2D-subtilisin M2-F  PK7WG2D-subtilisin M2-R  PK7WG2D-subtilisin M3-F  PK7WG2D-subtilisin M3-R  PK7WG2D-subtilisin M4-F  PK7WG2D-subtilisin M4-R  PK7WG2D-subtilisin M5-F  PK7WG2D-subtilisin M5-R  PK7WG2D-subtilisin M6-F  PK7WG2D-subtilisin M6-R  PK7WG2D-subtilisin M7-F  PK7WG2D-subtilisin M7-R  PK7WG2D-subtilisin M8-F  PK7WG2D-subtilisin M8-R  PK7WG2D-subtilisin M9-F  PK7WG2D-subtilisin M9-R  PK7WG2D-subtilisin M10-F  PK7WG2D-subtilisin M10-R  PK7WG2D-subtilisin M11-F  PK7WG2D-subtilisin M11-R  PK7WG2D-subtilisin M12-F  PK7WG2D-subtilisin M12-R  PK7WG2D-subtilisin M13-F  PK7WG2D-subtilisin M13-R | CAGGATGAGAAGAATCGATGGCGCTGGCGATAACCGCGGCTTTCACATTTGATCCAGTG  ATCGATTCTTCTCATCCTGATTTAAAAGTAGC  CCTGCTACTTTTAAATCAGGGGCAGAAGAATCGATACCGCTGTCG  CCTGATTTAAAAGTAGCAGG  TGAGTTCCGTGAGAGTTGTATGCTTGGAAAGGATTTGTTTCAG  TACAACTCTCACGGAACTCACGTTGCCGGTACAGT  GCAACTGTACCGGCAACGTGAGCTGCTGCAGAGTTGTAGTCTTGGAAAG  CACGTTGCCGGTACAGTTGCGGCTCTT  ACAGCGTAAAGAGATGCGCTAGCCGCAACGCCTAATACACCGA  AGCGCATCTCTTTACGCTGTAAAAGTTCTCGGCG  TGGCCGGAACCGTCAGCGCCTGCAGCTTTTACAGCGTAAAGAGATG  GGCGCTGACGGTTCCGGCCAGTAC  GCTGCAGAACCAGAAGGTCCGGCTGCTGCCATGTTAATAACGTCCATAT  GGACCTTCTGGTTCTGCAGCGTTAAAAGCGG  ATGACAGAAGGGTATTTACCTGCGTAGCCCACTGTGCTTGAGC  GGTAAATACCCTTCTGTCATTGCGGTAGGCGCTGT  TGGTTGCTGCTGTTAACAGCCGCCGCCGCAATGACAGAAGGGTATT  GCTGTTAACAGCAGCAACCAAAGAGCATCTTTCTCAAGCG  TGGTTGCTGCTGTTAACAGCCGCCGCCGCAATGACAGAAGGGTATT  GCTGTTAACAGCAGCAACCAAAGAGCATCTTTCTCAAGCG  GCCATGACATCAAGCTCAGACGCTACGCTCGCGAAAGATGCTCTTTGGTTGC  TCTGAGCTTGATGTCATGGCACCAGGCGTC  CGTGCTTTGGATAGAGACTGCTGGTGCCATGACTGCAAGCTCAGAACCTACGCT  GTCTCTATCCAAAGCACGCTTCCTGGAAACAAATACG  ACGTGCGGAGATGCCATTGATGCTGCATTGTACGCGCCGTATTTGT  TCAATGGCATCTCCGCACGT |

**Supplemental Table 5：Quantitative RT-PCR primers**

| Primer ID | Sequence |
| --- | --- |
| *actin-*F  *actin-*R  *WRKY7-*F  *WRKY7-*R  *WRKY8-*F  *WRKY8-*R  *Acre31-*F  *Acre31-*R  *Pti1-*F  *Pti1-*R  *CYP71D20-*F  *CYP71D20-*R  *FRK*-F  *FRK*-R | CGAGGGTTATGCTTTGCCTC  AGCAAGCTCCTCCTTCATGT  Cagcgcgtaaacatgtggaaagag  Ccaaagaagatctgagccgtggag  Cacaaccatccaaagccgactcag  Gggtctggttcttttgagtctggtt  Ccagccatccacgatccattcata  Gaagaagacttaagtttgcccctgg  Gacaggtcaagtgcgcttcttcg  Caatagttggtgcgctcgtggg  Accgcaccatgtccttagag  Cttgccccttgagtacttgc  AGCAGTTGACGGACAAAAGTG  TGTGTAGTTTTGCCCATTGG |
